# The Prevalence and Social Determinants of Hypertension among Adults in Indonesia: A Cross-Sectional Population-Based National Survey

**DOI:** 10.1155/2018/5610725

**Published:** 2018-08-09

**Authors:** Karl Peltzer, Supa Pengpid

**Affiliations:** ^1^HIV/AIDS/STIs and TB (HAST), Human Sciences Research Council, Pretoria 0001, South Africa; ^2^Department of Research and Innovation, University of Limpopo, Sovenga 0727, South Africa; ^3^ASEAN Institute for Health Development, Mahidol University, Salaya, Thailand

## Abstract

**Background:**

Hypertension is the most significant avoidable cause of morbidity and mortality, yet nationally representative adult data on Indonesia have not been available. The study aimed at assessing the prevalence and determinants of hypertension, including sociodemographic variables, weight status, health behaviour, and psychosocial stress and support risk factors.

**Methods:**

The Indonesia Family Life Survey (IFLS-5) interviewed and examined in a national population-based cross-sectional study 29965 individuals aged 18 years and older, mean age 43.3 years (SD=15.3). Blood pressure, body height and weight, dietary behaviour, physical activity, tobacco use, and psychosocial variables were measured. Logistic regression analyses were used to estimate determinants of hypertension by gender.

**Results:**

The prevalence of hypertension among study participants was 33.4 % (95 % CI: 32.7-34.0), among males 31.0% (95% CI: 30.2, 31.9), and among females 35.4% (95% CI: 34.6, 36.3). Among hypertensives, 42.9% were aware, 11.5% were treated, and 14.3% were controlled. In fully adjusted analyses, in both men and women, older age, no or elementary education, being overweight or obese, and having visited an outpatients health facility in the past 4 weeks were positively associated hypertension. Significant linear relationships of hypertension were found with age (*P* for trend <0.001) and body mass index (BMI) (*P* for trend < 0.001). In addition, among men having quit tobacco use and depressive symptoms were positively associated with hypertension, while current tobacco use was negatively associated with hypertension. Moreover, among women lower subjective economic status was associated with hypertension.

**Conclusions:**

The prevalence of hypertension was high and awareness was low, and treatment and control were very low. Significant multilevel public health interventions are urgently needed to improve the diagnosis, treatment, and control of hypertension in Indonesia.

## 1. Introduction

Chronic noncommunicable diseases (NCDs) have become an enormous health burden in low- and middle-income countries [1]. The scale of the increase of NCDs can be particularly observed in Southeast Asia, such as in Indonesia, where a limited budget has been allocated to combat the growing NCD epidemic [2]. The three top causes of death in Indonesia are stroke (21.2%), ischaemic heart disease (8.9%), and diabetes mellitus (6.5%) [3]. Major adult NCD risk factors in Indonesia include current tobacco smoking (in 2011) 35% (67% in men and 3% in women), high blood pressure or hypertension (in 2008) 27.8% (29.1% in men and 26.5% in women), and obesity (in 2008) 4.8% (2.6% in men and 6.0% in women) [4].

Hypertension is a preventable illness condition and has been found to be associated with an unhealthy lifestyle, including tobacco smoking, a lack of physical activity, and alcohol consumption [1]. Increasing trends in the prevalence of hypertension have been shown in high- and low- and middle-income countries [5, 6]. Comparable with the global trend, “the single most attributable cause for mortality in South-East Asia” is hypertension with an increasing trend [7]. Hussain et al. [8] found in the “2007 Indonesian Family Life Survey (IFLS-4)” a prevalence of 47.8% with hypertension in individuals 40 years and older. In other national population-based surveys in Southeast Asia the prevalence of hypertension was in Malaysia 43.5% (≥30 years in 2011) [9], in Myanmar 30.1% (15-64-year-olds in 2009) [10], in Sri Lanka 23.7% (≥18 years in 2005-2006) [11], and in Vietnam 25.1% (≥25 years in 2002-2008) [12], while in two other Asian countries the national prevalence of hypertension was in China 29.6% (≥18 years in 2010) [13] and in Iran 25% (25-64-year-olds in 2005) [14]. There is a lack of data on the adult (15 years and older) hypertension prevalence in Indonesia.

Hypertension is a preventable disease but recent studies found a low prevalence of antihypertensive medication treatment. In the 2007 IFLS4 survey (40-65 years) in Indonesia, 37% of individuals with hypertension were diagnosed or aware and 25% were treated with prescribed antihypertensive medication [8]. In the previously reported national population-based surveys in China (≥18 years and older), 42.6% were aware of their hypertension status and 34.1% received antihypertensive treatment [13], in Iran (25-64 year-olds) 34% were aware and 24.8% treated [14], and in Vietnam [≥25 years] 48.4% were aware and 29.5% received treatment [12]. In a community study with participants (35-70 years) from 17 mainly low- and middle-income countries, 46.5% were aware of their hypertension diagnosis and 32.5% were treated [15]. These studies indicate the very important need for the improvement of the diagnosis and treatment of hypertension [15].

Various risk factors have been found to be linked with hypertension, including sociodemographics (older age, female or male gender, lower education level, and lower household income) [8, 10–12, 14–17], geolocality (urban residence) [10, 12, 18], and other risk factors, including body weight status and health behaviour and psychosocial stress and support. Higher body mass index (BMI) has been found to be positively [8, 10, 13, 15–17] and underweight status negatively [10] associated with hypertension. Various dietary behaviours were also associated with hypertension, including insufficient fruit and vegetable intake [19–21], consumption of fatty foods [22, 23] and fast foods [24, 25], and sugar and soft drink consumption [26]. A number of studies found an association between physical inactivity [10, 13, 27–29], smoking [29, 30], and hypertension. Some studies, e.g., [31], found that a higher frequency of doctor visits was associated with having hypertension in addition to psychosocial stress [32, 33], including depression [34], low life satisfaction [35, 36], and less social cohesion [37] and lack of social support [32].

The aim of this study was to assess the prevalence of hypertension and to identify sociodemographic, weight status, health behaviour, psychosocial stress, and support risk factors for hypertension based on a cross-sectional survey of the IFLS-5 in 2014-15 in Indonesia.

## 2. Materials and Methods

### 2.1. Study Design and Participants

Data were analysed from the “Indonesia Family Life Survey (IFLS-5)”, a continuing demographic and health survey that began in 1993 and had since four rounds of data collection, with the fifth wave (IFLS-5) having been completed in 2015 [38]. The community surveys collected data on household, individual, and community level using a multistage stratified sampling [38]. The sampling frame of the first survey in 1993 was based on households from 321 enumeration areas (EAs) (20 households were randomly selected from each urban EA, and 30 households were selected from each rural EA) in 13 out of 27 Indonesian provinces that were selected representing 83% of the Indonesian population in 1993; more details are available in [38]. At household level, several randomly selected household members were asked to provide detailed individual information. In the IFLS-5, 16,204 households and 29965 18 years and older individuals were interviewed and had complete blood pressure measurements. In the IFLS-5, “the dynasty recontact rate was 92% and for the individual target households (including split off households as separate) the re-contact rate was 90.5%.” [39]. Although the survey is longitudinal, we restricted our analysis to the IFLS-5 because it was the only survey to include complete hypertension information for persons 18 years and older.

### 2.2. Measures

Measures included blood pressure measurements, anthropometric measurements, and questionnaire items on sociodemographic factors, dietary behaviour, physical activity, tobacco use, health care utilization, psychosocial stress, and support.

#### 2.2.1. Blood Pressure (BP) Measurements and Classification

Three successive measurements of systolic and diastolic blood pressure (BP) were recorded with an Omron meter, HEM-7203, by regular trained interviewers on household members 18 years and older at home in a seated position [38]. Mostly a normal sized cuff was used, while large cuffs were available when needed [38]. The first BP measurement was conducted at the onset of the research interview, with two subsequent assessments during the course of the interview [38]. Information was collected on awareness (having been diagnosed of hypertension by a doctor, nurse, paramedic, and trained mid-wife) and treatment of hypertension (currently taking prescribed medication on a weekly basis to manage your hypertension). The current analysis is restricted to individuals who had information on hypertension measurement, awareness, and treatment. For the three measurements of each systolic and diastolic blood pressure average blood pressure was calculated arithmetically.

Blood pressure was classified using JNC 7 algorithm [39]. “Hypertension was defined as systolic BP ≥140 mm Hg and/or DBP ≥90 mm Hg and/or current use of antihypertensive medication. Normotension was defined as BP values <120/80 mm Hg in individuals who were not taking antihypertensive medication” [39].

*Sociodemographic factors* were assessed with questions on age, gender, education, residential status, subjective socioeconomic background, and country region. Subjective economic status was assessed by the question “Please imagine a six-step ladder where on the bottom (the first step), stand the poorest people, and on the highest step (the sixth step), stand the richest people. On which [economic] step are you today?” The answers ranged from (1) poorest to (6) richest [38].

#### 2.2.2. Weight Status and Health Behaviour

*Anthropometric Measurements*. Heights were measured to the nearest millimetre using a Seca plastic height board (model 213) [38]. Weights were taken to the nearest tenth of a kilogram using a Camry model EB1003 scale [38]. Body mass index (BMI) was calculated as weight in kg divided by height in metre squared and classified according to Asian criteria: normal weight (18.5 to <23.0 kg/m^2^), overweight (23.0 to <25.0 kg/m^2^) and obese (25+ kg/m^2^) [40].

*Vegetable consumption *was measured with two items, “How many days in the past week, (1) Green leafy vegetables and (2) carrots had been eaten?” [38].

*Fruit consumption *was assessed with three questions on, “How many days in the past week, (1) Banana, (2) Mango and (3) Papaya had been eaten?” [38].

Additional dietary variables included “on how many days in the past week, (1) Fast food, (2) Fried snacks (tempe, tahu, bakwan, etc.), and (3) Soft drinks (Coca cola, sprite, etc.) had been consumed?” [38].

*Physical Activity*. Physical activity was assessed using a modified version of the International Physical Activity Questionnaire (IPAQ) short version, for the last 7 days (IPAQ-S7S). We used the instructions given in the IPAQ manual [41] and categorized physical activity according to the official IPAQ scoring protocol [42] as low, moderate, and high.

*Tobacco use *was assessed with two questions: (1) “Have you ever chewed tobacco, smoked a pipe, smoked self-enrolled cigarettes, or smoked cigarettes/cigars?” (Yes, No) and (2) “Do you still have the habit or have you totally quit?”(Still have, Quit) [38]. Responses were grouped into never, quitters, and current tobacco users.

*Health care utilization* was assessed with the question, “Whether visited any outpatient health care clinic in one month prior to survey or not?” (Yes, No) [38].

#### 2.2.3. Psychosocial Stress and Support

The* Centres for Epidemiologic Studies Depression Scale (CES-D: *10 items) was used to assess depressive symptoms, and scores 10 or more were classified as having depressive symptoms [43] (Cronbach's alpha = 0.71).

*Life satisfaction *was assessed with one item, “Please, think about your life as a whole. How satisfied are you with it?” Response option ranged from 1: completely satisfied to 5: not at all satisfied [38].

*Social support or capital *was assessed with 4 items (selected from 11 items through factor analysis), “During the last 12 months did you participate or use?...” (1) Community meeting, (2) Voluntary labour, (3), Programme to improve the village/neighbourhood, and (4) Religious activities. Response options were “yes” or “no” [38] (Cronbach's alpha 0.60).

### 2.3. Data Analysis

Descriptive statistics were used to describe the study variables of the study population and Chi-square test to calculate difference in proportions. Nonparametric tests were used for trend analyses across ordered groups. Logistic regression (forced entry) analyses were used to test significant determinants of hypertension status, with hypertension serving as the dichotomous outcome variable and in Model 1 age served as independent predictor variable and in Model 2 sociodemographics, health, nutrition, psychosocial stress, and social capital served as the independent predictor variables. We also examined linear associations by exposure categories in each model (p for trend) in the assessment of hypertension prevalence. “Cross-section analysis weights were applied to correct both for sample attrition from 1993 to 2014, and then to correct for the fact that the IFLS1 sample design included over-sampling in urban areas and off Java. The cross-section weights are matched to the 2014 Indonesian population, again in the 13 IFLS provinces, in order to make the attrition-adjusted IFLS sample representative of the 2014 Indonesian population in those provinces”[38]. Both the 95% confidence intervals and P values were adjusted considering the survey design of the study. All analyses were conducted with STATA software version 13.0 (Stata Corporation, College Station, TX, USA).

## 3. Results

### 3.1. Sample Characteristics

The total sample included 29965 individuals 18 years and older, with complete hypertension measures (females: 52.7%; mean age 43.3 years, SD: 15.3, age range of 18–109 years) from Indonesia. Overall, 33.4% of the population had hypertension, 31.0% among men and 35.4% among women. Among hypertensives 42.9% were aware that they had hypertension, which was significantly higher in women (50.1%) than men (33.7%) and in urban (44.5%) than rural areas (41.1%). Of the population with hypertension, 11.5% were currently using prescribed antihypertensive medication, and 14.3% had controlled their blood pressure (<140 mm Hg and diastolic blood pressure <90 mm Hg). Women (14.0%) were more often than men (8.4%) and urban dwellers (13.0%) more often than rural dwellers (9.9%) using currently prescribed antihypertensive medication. Of those participants with hypertension, only 14.3% controlled their blood pressure. Mean systolic blood pressure was 1.1 mmHg higher for men than for women, while mean diastolic blood pressure was 0.2 mmHg higher for women than for men. The prevalence of BMI obesity was higher in women (42.0%) than in men (23.0%), while the current tobacco use prevalence was significantly higher in men (67.2%) than in women (3.0%) (see Table 1).

The prevalence of hypertension increases overall and for both sexes by age group from 18 to 29 years to 70 years and above (*P* for trend <0.001). The prevalence of hypertension is higher among males than females at the age 18 to 29 years, while from 40 years and older the prevalence of hypertension is higher among women than men. Further the prevalence of hypertension decreases overall and for both sexes (and stronger for females) by education from having no education to having higher education (*P* for trend <0.001) (see Table 2).

## 4. Association between Psychosocial Risk Factors and Hypertension

Table 3 shows associations (odds ratios) between independent variables and the prevalence of hypertension, in Model 1 adjusted for age, and in Model 2 adjusted for all covariates in the table. In fully adjusted models in both men and women, older age, no or elementary education, being overweight or obese, and having visited an outpatients health facility in the past 4 weeks were positively associated with hypertension. Both among men and among women being underweight was negatively associated with hypertension. In addition, among men having quit tobacco use and depressive symptoms were positively associated with hypertension, while current tobacco use was negatively associated with hypertension. Moreover, among women lower subjective economic status was associated with hypertension. In Model 1, the prevalence of hypertension was higher among men in urban than rural areas, while this became insignificant in Model 2, the fully adjusted model. Significant positive linear relationships of hypertension were found among both men and women with age (P<0.001) and BMI (P<0.001), and negative relationships among men with tobacco use (P=0.006) and among women with economic background (P=0.015) (see Table 3).

## 5. Discussion

In this first nationally representative population-based survey on hypertension in Indonesia, the age-adjusted prevalence in all adults (18 years and older) was 33.4% (48.6% in 40 years and older) in 2014-15, which is similar to the prevalence among 40 years and older in the 2007 survey in Indonesia (47.8%) [8], the Myanmar survey in 2009 of 30.1% in 15-64-year-olds [10], and the China survey in 2010 of 29.6% in individuals 18 years and older [13], and similar to the global adult (≥20 years) prevalence in low- and middle-income countries (31.5%) [44], but higher than in Iran [14, 45], Sri Lanka [11], and Vietnam [12].

Of those who had hypertension only 42.9% were aware (44.9% among 40 years and older) and 11.5% were using prescribed antihypertensive medication (13.8% among 40 years and older) in this 2014-15 survey. This seems to show an increase in the awareness (from 37% in 2007 to 44.9% in 2014-15) but a decrease in the proportion of treatment (from 25% in 2007 to 11.5% in 2014-15) [8]. The proportion of awareness in this study was also similar to figures reported from China [13], Vietnam [12], and globally in low- and middle-income countries [15, 45]; however, the proportion of reported treatment was in this study significantly lower than in other countries, including China [13], Iran [14], Vietnam [12], and globally in low- and middle-income countries studies [15, 45]. The awareness and treatment of hypertension was in this study greater among women than men, as also found previously [8]. The awareness and treatment of hypertension was higher in urban than rural areas. This may be because of lack of access to health care services in rural areas [8]. The increase in the awareness of hypertension may be attributed to community-based integrated coaching posts that enable community participation in the early detection of hypertension [46]. These findings seem to suggest that despite some improvement in the awareness of hypertension more should be achieved, in particular among men, and appropriate treatment needs to be seriously improved, especially also among men and in rural areas.

This study found, in agreement with various studies [8, 10–12, 14–17], that older age and lower educational level were among both men and women associated with hypertension. Lower subjective economic status was among women but not among men associated with hypertension. Although the prevalence of hypertension increased overall and for both sexes by age group from 18 to 29 years to 70 years and above, the prevalence of hypertension was higher among males than females at the age 18 to 29 years, while from 40 years and older the prevalence of hypertension was higher among women than men. One possible reason for this age differential among the sexes can be because the prevalence rates of obesity, as one important risk factor for hypertension, are higher in boys than girls among children, adolescents, and young adults [47–49] but higher in females in the adult age groups [49]. The high prevalence of hypertension in the group with low or no education in this study may be related to having a higher number of risk factors such as stress, poor dietary habits, poor working conditions, and lack of access to health services [50]. Urban residence increased the odds for hypertension in previous studies [10, 12, 18] but in this study it was only significant in bivariate analysis among men and nonsignificant for women. It seems that the urban-rural differences in the prevalence of hypertension, as found in the 2007 IFLS [8], no longer exist in the 2013-14 IFLS in Indonesia.

Regarding weight variables, being underweight was protective and being overweight or obese increased the odds of having hypertension. The association between overweight/obesity and hypertension has been confirmed in a number of studies [8, 10, 13, 15–17]. Overweight or obesity may be associated with hypertension independently but it could also be mediated through unhealthy diet and inadequate physical activity [14]. The negative association between being underweight and hypertension was also found in a study in Myanmar [10]. Some studies [e.g., [51]] suggest that about two-thirds of the prevalence of hypertension may be attributed to obesity.

As found in a number of previous studies [10, 13, 27–29], moderate physical activity was in this study among men but not among women negatively associated with hypertension. The elevated blood pressure among the female participants of the study may not be attributable to physical inactivity but linked to other factors such as obesity [52]. In agreement with some previous studies [22, 23], this study found that consumption of fatty foods (fried snacks) was among women associated with hypertension.

Unlike previous studies [19–21, 24–26], this study did not find an association between fruit and vegetable, soft drink, fatty foods (fried snacks), fast food consumption, and hypertension. Current smoking is a significant risk factor for hypertension [29, 30]. However, in this study among men only former tobacco use was positively associated with hypertension, while current tobacco use was negatively associated with hypertension. Among smokers in this study the number of cigarettes smoked per day (Mean=11.7) did not differ between hypertensives and nonhypertensives (Mean: 11.9) (P=0.150). It is possible that the impact of current tobacco use on hypertension is delayed, and, thus, current tobacco use may not be closely correlated with hypertension [53]. In a life-course impact of smoking on hypertension study, Gao et al. [53] found that current number of cigarettes smoked per day was negatively associated with the risk of hypertension; however, the increase of life-course-adjusted number of cigarettes smoked per day was associated with higher risks of hypertension.

The burden of having hypertension may also be expressed in the association between past 4 weeks outpatient health care utilization and hypertension, as also found in a previous study [31] of hypertension. In terms of psychosocial stress and support, this study found, in agreement with previous studies [32–34], that depression was among men (and in bivariate analysis among women) positively related to hypertension. Hyperreactivity of the sympathetic nervous system and genetic influences seem to be influencing mechanisms in the relationship between depression and hypertension [54]. Unlike previous studies [32, 35–37], this study did not find an association between low life satisfaction and lack of social support or capital and hypertension.

## 6. Study Limitations

Apart from anthropometric and blood pressure measurements, a study limitation was that all the other information assessed in this analysis was based on self-reporting. It is possible that certain behaviours were over- or underreported. Several essential determinants for hypertension such as serum fasting blood surge, Low-Density Lipoprotein (LDL), and total cholesterol (TC) were not assessed in this study and should be included in future studies. Further, it was a cross-sectional study and causal relationships between risk factors and the development of hypertension can be established. Another limitation was that salt intake and heavy alcohol use, which are determinants of hypertension, were not assessed in this study.

## 7. Conclusion

The study found a high prevalence of hypertension in a representative sample of the general adult population in Indonesia. Less than half of hypertensives were aware and a minority were treated and controlled. Several risk factors, including sociodemographic variables (older age, lower education, being female, and lower socioeconomic status), body weight status (overweight or obese), health behaviour (physical inactivity), and psychosocial stress (depression), have been identified, which can help in guiding intervention programmes. Interventions programmes operating at multiple levels are urgently needed that can increase awareness of hypertension, access to high blood pressure treatment, and community wide health behaviour interventions that were identified and known to be effective in reducing high blood pressure levels. Future studies may want to include additional measures such as salt intake and alcohol consumption.

## Figures and Tables

**Table 1:** Sample characteristics.

Variable name	Variable specification	Total	Male	Female	P value
Sample	N (%)	29965	13997 (47.3)	15969 (52.7)	

Mean age in years (SD)	Range 18-109, mean (SD)	43.3 (15.3)	42.0 (15.3)	42.6 (15.2)	0.056

Systolic blood pressure	mmHg, mean (SD)	130.9 (21.9)	131.8 (19.2)	130.1 (24.1)	<0.001

Diastolic blood pressure	mmHg, mean (SD)	80.1 (11.9)	80.0 (11.7)	80.2 (12.1)	0.004

Hypertension	%	33.4	31.0	35.4	<0.001

Of hypertensives	Aware, %	42.9	33.7	50.1	<0.001

Of hypertensives	Treated, %	11.5	8.4	14.0	<0.001

Of hypertensives	Controlled, %	14.3	12.4	15.7	<0.001

Education	None/elementary, %	44.0	39.1	48.4	<0.001

Subjective economic background	Poor, % Medium, % Rich, %	25.5 46.6 27.9	27.4 48.9 23.7	23.8 44.6 31.6	<0.001

Residence	Urban, %	58.6	58.6	58.7	0.198

Region	Sumatra, % Java, % Main islands^1^, %	22.7 54.6 22.7	23.4 54.2 22.4	22.1 54.9 23.0	0.027

**Body weight status and health behaviour**					

Body Mass Index (BMI)	Normal, % Underweight, % Overweight, % Obese, %	40.2 11.2 16.0 32.4	48.3 13.5 15.2 23.0	33.2 9.0 16.8 42.0	<0.001

Vegetable consumption^2^	<7 days/week, %	57.6	58.3	56.7	<0.001

Fruit consumption^3^	<3 days/week, %	58.1	59.6	56.8	<0.001

Fast food	≥once week, %	10.6	9.8	11.2	0.033

Fried snacks	4-7 times/week, %	25.6	26.3	25.3	0.147

Soft drinks	≥once week, %	18.5	24.9	12.8	<0.001

Physical activity	Low, % Moderate, % High, %	48.3 26.6 25.1	43.1 22.9 34.0	49.7 30.0 20.3	<0.001

Tobacco use	Never, % Quit, % Current, %	62.2 5.0 32.8	23.2 9.6 67.2	95.9 1.1 3.0	<0.001

Health care utilization	Past 4 weeks, %	18.8	14.6	23.9	<0.001

**Psychosocial stress and support**					

Depression symptoms	Scores ≥10, %	22.8	22.2	23.4	0.177

Life satisfaction	High-yes, %	42.7	40.2	44.9	<0.001

Social support/capital	High-yes, %	37.9	50.6	26.4	<0.001

^1^Major island groups: Bali, West Nusa Tenggara, South Kalimantan, and South Sulawesi; ^2^vegetable: green leafy vegetables and/or carrots; ^3^fruits: banana and/or mango and/or papaya.

**Table 2:** Prevalence of hypertension among individuals 18 years and older in Indonesia.

Variable	Total sample	Male		Female		Total	
	N	n	% (95% CI)	N	% (95% CI)	N	% (95% CI)

All	29965	4040	31.0 (30.2, 31.9)	4925	35.4 (34.6, 36.3)	8965	33.4 (32.7, 34.0)

Age group							

18-29	8488	535	13.9 (12.8, 15.2)	433	9.3 (8.4, 10.2)	968	11.6 (10.9, 12.4)

30-39	8444	804	19.2 (17.8, 20.5)	834	19.5 (18.2, 20.9)	1638	19.3 (18.4, 20.3)

40-49	5586	874	31.3 (29.5, 33.2)	1093	39.1 (37.2, 41.1)	1967	35.6 (34.3, 37.0)

50-59	3834	809	45.6 (43.1, 48.2)	1167	56.7 (54.4, 59.0)	1967	51.3 (49.5, 53.0)

60-69	2105	582	58.1 (54.7, 61.4)	730	66.0 (62.8, 69.0)	1312	62.1 (59.8, 64.4)

70 or more	1508	436	67.1 (63.1, 70.9)	668	79.3 (76.0, 82.3)	1104	74.1 (71.5, 76.4)

			*P-* trend <0.001		*P-* trend <0.001		*P-* trend <0.001

Education							

None	1568	200	47.2 (41.8, 52.6)	699	59.3 (56.0, 62.5)	899	56.1 (53.2, 58.8)

Elementary	9741	1525	36.8 (35.2, 38.4)	2292	45.2 (43.8, 46.7)	3817	41.5 (40.4, 42.6)

High school	14305	1736	25.7 (24.6, 26.9)	1537	24.6 (23.5, 25.8)	3273	25.2 (24.4, 26.0)

Higher education	4294	567	30.1 (27.9, 32.5)	365	20.0 (18.1, 22.1)	932	25.1 (23.4, 26.6)

			*P*- trend <0.001		*P-* trend <0.001		*P-* trend <0.001

**Table 3:** Predictors of hypertension by gender.

**Variable**	**Male**		**Female**	
	Age-adjusted (95% CI)^a^	AOR (95% CI)^b^	Age-adjusted (95% CI)^a^	AOR (95% CI)^b^

**Sociodemographics**				

Education				
High school or Higher education	1 (Reference)	1 (Reference)	1 (Reference)	1 (Reference)
None or elementary	1.13 (1.03, 1.25)*∗∗*	1.15 (1.02, 1.28)*∗*	1.34 (1.22, 1.47)*∗∗∗*	1.39 (1.25, 1.54)*∗∗∗*

Subjective economic background				
Poor	1 (Reference)	1 (Reference)	1 (Reference)	1 (Reference)
Medium	1.06 (0.95, 1.18)	0.94 (0.84, 1.05)	0.87 (0.78, 0.97)*∗*	0.89 (0.79, 0.99)*∗*
Rich	1.18 (1.04, 1.34)*∗∗*	0.98 (0.85, 1.12)	0.82 (0.73, 0.93)*∗∗*	0.85 (0.75, 0.96)*∗*
*P* for trend	0.011	0.842	0.002	0.015

Residence				
Rural	1 (Reference)	1 (Reference)	1 (Reference)	1 (Reference)
Urban	1.17 (1.07, 1.28)*∗∗∗*	1.02 (0.92, 1.12)	1.07 (0.98, 1.16)	1.08 (0.98, 1.18)

Region				
Sumatra	1 (Reference)	1 (Reference)	1 (Reference)	1 (Reference)
Java	0.97 (0.88, 1.08)	0.96 (0.86, 1.07)	1.04 (0.94, 1.15)	0.98 (0.88, 1.10)
Main islands	1.06 (0.94, 1.20)	1.03 (0.90, 1.17)	1.06 (0.94, 1.20)	1.11 (0.97, 1.26)

**Body weight status and health behaviour**				

Body Mass Index (BMI)				
Underweight	0.66 (0.56, 0.78)*∗∗∗*	0.63 (0.53, 0.74)*∗∗∗*	0.72 (0.60, 0.86)*∗∗∗*	0.69 (0.57, 0.84)*∗∗∗*
Normal	1 (Reference)	1 (Reference)	1 (Reference)	1 (Reference)
Overweight	1.93 (1.70, 2.20)*∗∗∗*	1.99 (1.74, 2.26)*∗∗∗*	1.29 (1.13, 1.48)*∗∗∗*	1.32 (1.15, 1.51)*∗∗∗*
Obese	3.01 (2.70, 3.36)*∗∗∗*	3.02 (2.69, 3.39)*∗∗∗*	2.18 (1.96, 2.43)*∗∗∗*	2.24 (2.01, 2.50)*∗∗∗*
*P* for trend	<0.001	<0.001	<0.001	<0.001

Vegetable consumption less than daily (base=daily)	0.92 (0.84, 1.01)	0.94 (0.86, 1.04)	1.07 (0.98, 1.16)	1.03 (0.93, 1.12)

Fruit consumption <trice/week (base=trice or more)	0.90 (0.82, 0.99)*∗*	0.99 (0.90, 1.09)	1.02 (0.93, 1.11)	1.04 (0.94, 1.14)

Fast food (once or more/week) (base= <once)	1.12 (0.96, 1.31)	0.96 (0.81, 1.14)	0.99 (0.85, 1.14)	1.00 (0.86, 1.17)

Fried snacks (4-7 times/week) (base=0-3 times)	1.06 (0.96, 1.18)	1.04 (0.93, 1.15)	1.12 (1.02, 1.24)*∗*	1.10 (0.99, 1.21)

Soft drinks (once or more/week) (base= <once)	1.12 (1.00, 1.25)*∗*	1.05 (0.94, 1.18)	0.97 (0.84, 1.11)	0.97 (0.84, 1.12)

Physical activity				
Low	1 (Reference)	1 (Reference)	1 (Reference)	1 (Reference)
Moderate	1.18 (1.05, 1.32)*∗∗*	1.20 (1.07, 1.40)*∗∗*	1.02 (0.92, 1.13)	1.01 (0.91, 1.11)
High	0.86 (0.78, 0.96)*∗∗*	0.95 (0.85, 1.06)	1.00 (0.89, 1.12)	1.00 (0.88, 1.12)
*P* for trend	0.011	0.487	0.945	0.951

Tobacco use				
Never	1 (Reference)	1 (Reference)	1 (Reference)	1 (Reference)
Quit	1.22 (1.04, 1.43)*∗*	1.19 (1.00, 1.40)*∗*	1.43 (0.94, 2.16)	1.56 (0.98, 2.48)
Current	0.74 (0.66, 0.82)*∗∗∗*	0.88 (0.79, 0.99)*∗*	0.92 (0.73, 1.17)	1.00 (0.78, 1.29)

Health care utilization	1.25 (1.11, 1.41)*∗∗∗*	1.16 (1.02, 1.32)*∗*	1.23 (1.12, 1.36)*∗∗∗*	1.22 (1.10, 1.35)*∗∗∗*

**Psychosocial stress and support**				

Depression symptoms	1.12 (1.01, 1.24)*∗*	1.17 (1.04, 1.31)*∗∗*	1.12 (1.01, 1.24)*∗*	1.09 (0.98, 1.21)

Life satisfaction (high) (base=low)	0.96 (0.88, 1.06)	0.91 (0.83, 1.01)	0.97 (0.89, 1.06)	0.97 (0.89, 1.06)

Social support/capital (high) (base=low)	0.97 (0.88, 1.06)	0.95 (0.87, 1.05)	0.92 (0.83, 1.01)	0.93 (0.83, 1.02)

AOR: Adjusted Odds Ratio; CI: Confidence Interval; ^a^adjusted for age; ^b^adjusted for all covariates; *∗∗∗* represents P<.001; *∗∗* represents P<.01; *∗* represents P<.05.

## Data Availability

The data underlying this study belong to the Indonesia Family Life Survey and are accessible via the RAND website: http://www.rand.org/labor/FLS/IFLS.html.

## Abbreviations

BMI: Body mass index
DBP: Diastolic blood pressure
CES-D: Centres for Epidemiologic Studies Depression Scale
EA: Enumeration area
IFLS: Indonesian Family Life Survey
IPAQ: International Physical Activity Questionnaire
LDL: Low-density lipoprotein
NCD: Noncommunicable disease
SBP: Systolic blood pressure.
